# Impact of Formalin- and Cryofixation on Raman Spectra of Human Tissues and Strategies for Tumor Bank Inclusion

**DOI:** 10.3390/molecules29051167

**Published:** 2024-03-06

**Authors:** Giulia Mirizzi, Finn Jelke, Michel Pilot, Karoline Klein, Gilbert Georg Klamminger, Jean-Jacques Gérardy, Marily Theodoropoulou, Laurent Mombaerts, Andreas Husch, Michel Mittelbronn, Frank Hertel, Felix Bruno Kleine Borgmann

**Affiliations:** 1 National Department of Neurosurgery, Centre Hospitalier de Luxembourg (CHL), 1210 Luxembourg, Luxembourg; 2 Saarland University Medical Center and Faculty of Medicine, 66421 Homburg, Germany; 3 Department of Cancer Research (DoCR), Luxembourg Institute of Health (LIH), 1445 Strassen, Luxembourg; 4 Department of Medicine IV, LMU University Hospital, LMU Munich, 80539 Munich, Germany; 5 Department of General and Special Pathology, Saarland University Medical Center (UKS), Saarland University (USAAR), 66424 Homburg, Germany; 6 National Center of Pathology (NCP), Laboratoire National de Santé (LNS), 3555 Dudelange, Luxembourg; 7 Luxembourg Center of Neuropathology (LCNP), 3555 Dudelange, Luxembourg; 8 Luxembourg Centre for Systems Biomedicine (LCSB), University of Luxembourg (UL), 4365 Esch-sur-Alzette, Luxembourg; 9 Department of Life Science and Medicine (DLSM), University of Luxembourg (UL), 4365 Esch-sur-Alzette, Luxembourg; 10 Hôpitaux Robert Schuman, 2540 Luxembourg, Luxembourg

**Keywords:** Raman spectroscopy, machine learning, tumor bank, formalin fixation, cryopreservation

## Abstract

Reliable training of Raman spectra-based tumor classifiers relies on a substantial sample pool. This study explores the impact of cryofixation (CF) and formalin fixation (FF) on Raman spectra using samples from surgery sites and a tumor bank. A robotic Raman spectrometer scans samples prior to the neuropathological analysis. CF samples showed no significant spectral deviations, appearance, or disappearance of peaks, but an intensity reduction during freezing and subsequent recovery during the thawing process. In contrast, FF induces sustained spectral alterations depending on molecular composition, albeit with good signal-to-noise ratio preservation. These observations are also reflected in the varying dual-class classifier performance, initially trained on native, unfixed samples: The Matthews correlation coefficient is 81.0% for CF and 58.6% for FF meningioma and dura mater. Training on spectral differences between original FF and pure formalin spectra substantially improves FF samples’ classifier performance (74.2%). CF is suitable for training global multiclass classifiers due to its consistent spectrum shape despite intensity reduction. FF introduces changes in peak relationships while preserving the signal-to-noise ratio, making it more suitable for dual-class classification, such as distinguishing between healthy and malignant tissues. Pure formalin spectrum subtraction represents a possible method for mathematical elimination of the FF influence. These findings enable retrospective analysis of processed samples, enhancing pathological work and expanding machine learning techniques.

## 1. Introduction

Recently, the technique of Raman spectroscopy (RS) has attracted interest in various research fields, ranging from the pharmaceutical sector to medical tumor segmentation [1,2,3]. RS is a label-free and user-friendly analytical technique that provides insights into the biochemical composition of a sample. This sustains the idea of introducing RS into the analysis and classification of human tissue samples. In our project, human brain tumor samples or other tissue samples extracted during neurosurgery were used. RS can rapidly scan tissues, and the potential for intraoperative use in neurosurgery suggests its promise as a valuable tool for future tissue and tumor identification. In the contemporary medical landscape, pinpointing the precise tumor type during surgery, in terms of quick section analysis, necessitates the extraction of a small sample, followed by fixation, embedding, cutting, and analysis by specialized professionals—a process that is both time-consuming and intricate [4,5,6,7]. Introducing intraoperative identification with RS would not only accelerate the procedure and reduce complexity but also potentially empower a surgical team member to undertake the task of preliminary tissue categorization prior to the detailed neuropathological analysis. 

To establish a systematic approach to integrating RS into tumor diagnostics, it is crucial to compile data from diverse tumor types and tissue samples. A feasible method involves accessing tissue samples sourced from tumor banks. In these repositories, tumors are either preserved by formalin fixation (FF) or cryopreservation (CP), thereby creating a highly valuable resource of well-preserved tumor specimens. These preserved samples can subsequently be retrieved for ongoing research and retrospective analysis of tumor characteristics, potentially leading to improved and more precise tumor therapy.

In order to assess the impact of fixation-induced alterations on machine learning procedures, we employed an internally developed two-class classifier. This classifier, with a sensitivity of 96.06 ± 0.03% and a specificity of 95.44 ± 0.02%, was proficient in distinguishing between native (NAT) meningioma and dura mater samples [8]. We then applied this classifier to both formalin-fixed (FF) and cryofixed (CF) samples for evaluation. Notably, there is currently no existing classifier capable of identifying both native and pathologically fixed samples in a single procedure. 

In this study, we focused on simple fixation methods, either chemically with formalin or physically by freezing with dry ice. Formalin fixation and paraffin embedding (FFPE) have been extensively examined in prior research and are known to cause substantial, lasting, and irreversible changes during the embedding process [9,10].

Formalin is a highly reactive solution characterized by its elevated electrophilic properties, which enable the long-term preservation of a sample’s biochemical composition. Formalin engages with various functional groups through cross-linking interactions. Its remarkable attributes include the prevention of decomposition, putrefaction, and autolysis, qualities that have made it a suitable choice for sample preservation since its initial use by Blum in 1892 [11,12].

Another widely employed method for preserving tissue samples is the practice of cryopreservation using dry ice. This technique is commonly applied in fields such as embryology, where it is used for preserving spermatocytes and oocytes, as well as in pathology for storing tumor samples in centralized banks, facilitating subsequent retrospective analysis of tissue characteristics [13,14,15,16]. Cryopreservation can be accomplished using either liquid nitrogen at −170 °C or dry ice at −80 °C, respectively, by putting the samples in a freezer at very low temperatures. 

In this investigation, we examine the Raman spectra of various sample entities, ranging from glioblastoma tissue to metastases and central nervous system (CNS) tissue. Additionally, we include samples of snap-frozen (dry ice) non-functioning pituitary adenomas (NFPA) that have been stored at −80 °C to assess their potential utility.

## 2. Results

**Figure 1:**
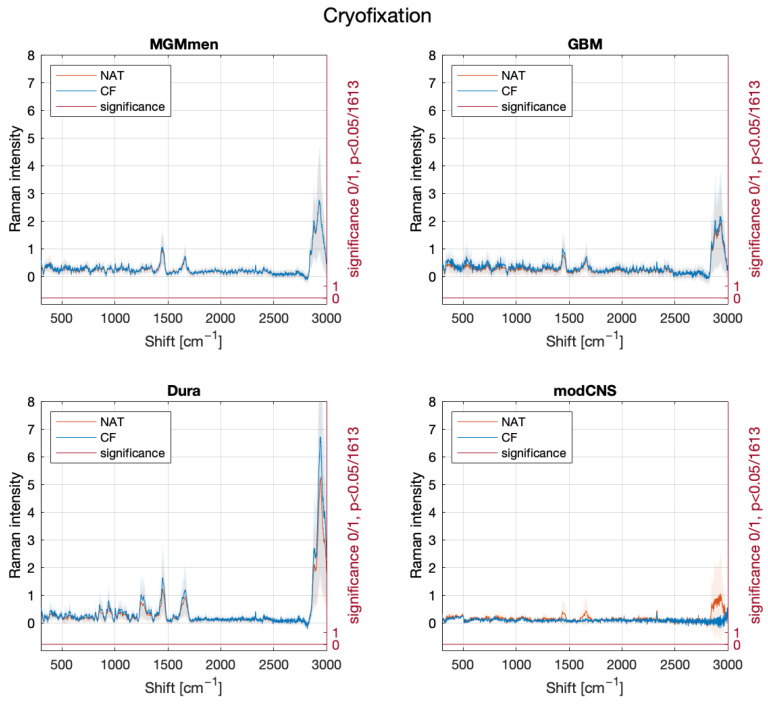
Mean spectra of the sample measurements in native and cryofixed conditions, with separation significance indicated on the bottom row in red. The significance at each single Raman shift (0 = null hypothesis, 1 = alternative hypothesis) is calculated using the Wilcoxon-Mann-Whitney-U test, and the *p*-value is Bonferroni-corrected (0.05/1613). This visualization reveals no discernible distinction in the spectra between the two conditions. Meningothelial meningioma (*MGM_men*, 31 native spectra and 21 CF spectra), dura mater (*Dura*, 24 native spectra and 12 CF spectra), glioblastoma (*GBM*, 38 native spectra and 22 CF spectra), modified CNS tissue (*modCNS*, 3 native spectra and 2 CF spectra) Note that both native and frozen samples gave very low intensity spectra compared to other specimens, likely due to the optical properties of the particular specimen. Modified CNS tissue is a tumor surrounding healthy brain tissue, revealing hypoxic and hemorrhagic changes.

**Figure 2:**
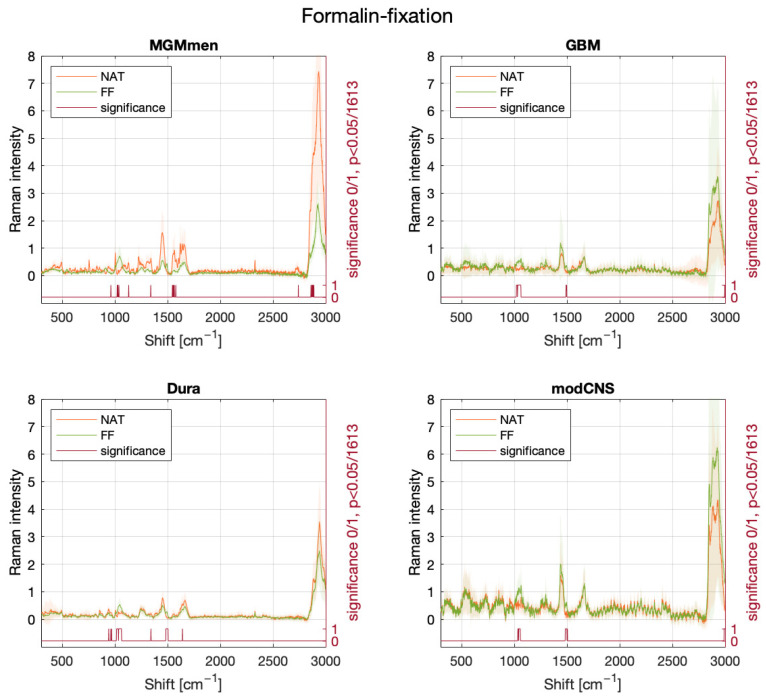
Mean spectra of the sample measurements in native and formalin-fixed conditions, with significantly different wavenumbers indicated on the bottom row in red. The significance at each single Raman shift (0 = null hypothesis, 1 = alternative hypothesis) is calculated using the Wilcoxon-Mann-Whitney-U test, and the *p*-value is Bonferroni-corrected (0.05/1613). As can be seen on the significant difference scale, formalin-fixation induces changes in the Raman spectrum at different sites for the different tissue entities depending on the molecular composition of the samples. Meningothelial meningioma (*MGM_men*, 13 native spectra and 17 FF spectra), dura mater (*Dura*, 25 native spectra and 43 FF spectra), glioblastoma (*GBM*, 49 native spectra and 24 FF spectra), and modified CNS tissue (*modCNS*, 32 native spectra and 51 FF spectra).

**Figure 3:**
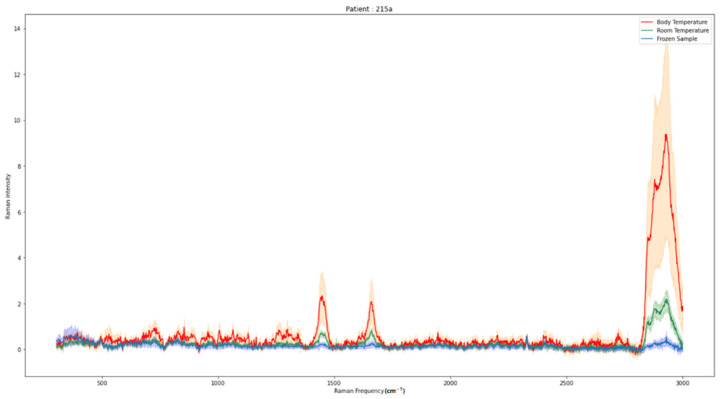
NFPA extracted from a tumor bank is first cryofixed (mean spectrum in blue, 309 spectra), then thawed (mean spectrum in green, 240 spectra), and heated at body temperature (mean spectrum in red, 80 spectra).

In the initial phase of our study, our focus lied on examining the potential interference of different fixation methods with the RS spectrum. Specifically, we investigated whether RS can discern between various fixation states and still recognize the sample entity. The emergence or disappearance of peaks and changes in intensity were investigated. We proposed two hypotheses. Our first hypothesis was that cryofixation with dry ice would not reveal new peaks but would result in an overall decrease in intensity [17]. Additionally, formalin fixation was expected to induce specific peaks with alterations in position and intensity based on the underlying sample’s biochemical structure [18]. Figure 1 shows no significant changes in the mean spectra between native and frozen samples, supporting our hypothesis. However, an overall decrease in intensity was observed in the cryofixed samples. Conversely, Figure 2 shows multiple spectral changes between native and formalin-fixed samples, in line with our second hypothesis. Figure 3 validates the cryofixation hypothesis by comparing the Raman spectrum of cryofixed NFPA, extracted from long-term storage at −80 °C, with the spectrum of thawed samples, revealing a reversibility of the overall intensity decrease. This decrease was also evident when measuring pure substances (pure water and formalin) initially in their native state and then frozen with dry ice (Supplementary Figure S1).

To validate these findings, we aimed to investigate whether a classifier, previously trained in a study comparing meningioma and dura mater, can maintain the same sensitivity and specificity when distinguishing meningioma and dura mater samples under fixed conditions. We assessed the discriminative capability of RS in differentiating the same meningioma sample scanned in its native state (immediately after neurosurgical extraction) and minutes later after freezing with dry ice, as outlined in the method section. In the native data set, 31 spectra for MGM and 24 spectra for dura mater were used. In the cryofixed section, we used 21 spectra for MGM and 12 spectra for dura mater. In a subsequent step, we repeated the process with a native meningioma (13 spectra) and dura mater (25 spectra) sample to differentiate between formalin-fixed meningioma (17 spectra) and dura mater (43 spectra). In a prior publication, we established that our meningioma-dura mater classifier, trained on native data, is a reliable tool for accurately detecting meningioma samples [8]. In this context, the same classifier is employed to analyze the extent to which it can recognize a meningioma or dura mater sample, even under frozen and formalin-fixed conditions.

**Figure 4:**
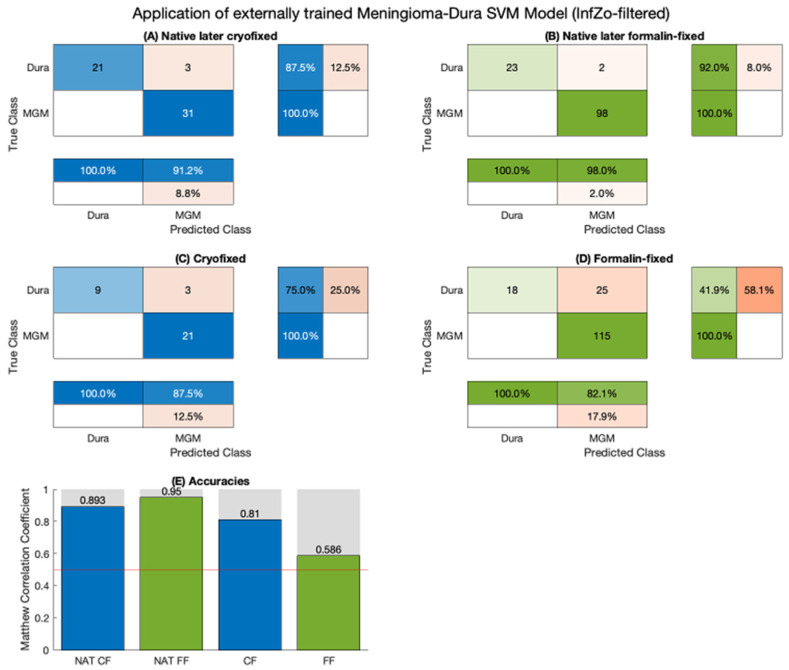
Applying an externally trained dual-class Support Vector Machine classifier to the samples under native and various fixed conditions, where cryo-fixed is labelled blue, formalin fixed green. The classification of meningioma and dura mater samples in native (**A**) and the classification in cryofixed conditions (**C**) reveals similar performance. In contrast, the classification of formalin fixed samples diminishes the classifier accuracy (**D**) compared to the native conditions (**B**) classifier results. (**E**) Overview comparing the classifier Matthews’ correlation coefficients.

Notable classifier performance was evident in distinguishing between cryofixed meningioma and dura mater. A classifier exclusively trained on native data exhibits the potential for effectively differentiating cryofixed samples without requiring additional training (Matthews’ correlation coefficient (MCC) 81.0%). However, this feasibility is not applicable to formalin fixation. As illustrated in Figure 4, classifier results are compromised when applied to formalin-fixed samples (MCC 58.6%). 

**Figure 5:**
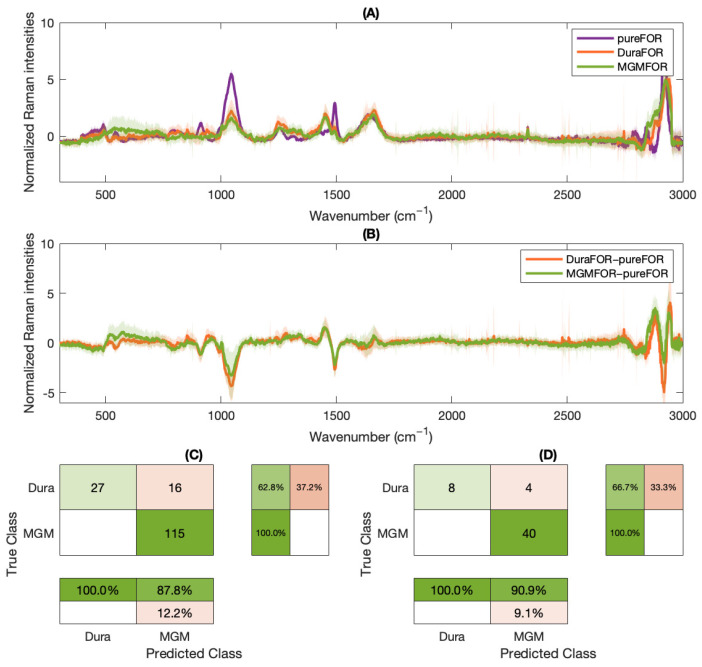
(**A**) Mean spectra of either formalin-fixed dura mater (DuraFOR, orange) or meningioma (MGMFOR, green), as well as pure-formalin (pureFOR, purple). (**B**) Differential spectrum obtained by subtracting the pure formalin spectrum from the average formalin-fixed dura mater (DuraFOR-pureFOR) or meningioma spectrum (MGMFOR-pureFOR), respectively. (**C**) Descriptive statistics after application of the externally trained meningioma/dura mater classifier on the differential spectra (MCC 74.2%). (**D**) Output of a separately trained classifier based on the subtracted spectra (MCC 77.9%). Validation was performed on an external data set.

Subsequently, our investigation aimed to determine whether enhancing the classifier results for formalin fixed samples is achievable by subtracting the pure formalin spectrum from their fixed spectrum when analyzed using a classifier trained on native data (Figure 5). An application of the classifier on the subtraction spectrum resulted in a slight improvement of the classifier performance on FF samples (MCC 74.2%), Figure 5C. This improvement was even enhanced after training a new meningioma-dura classifier with the same algorithm as in [8] to a MCC of 77.9%. Thereby, the validation was performed on an external data set, as shown in Figure 5D.

## 3. Discussion

Numerous authors have previously explored the biochemical changes and associated modifications in RS spectra resulting from tissue sample preservation methods, including cryo- and formalin-fixation [18,19,20,21,22,23,24]. 

The examination of freezing stages using RS has been documented in various literature sources, spanning from detailing the specific orientation of ice crystals to meticulously observing individual freezing stages discerned by variations in biochemical structure [19,20,21]. To our knowledge, RS has not been employed for the direct differentiation of native and fixed CNS tissues, nor has its potential for tumor entity recognition in cryopreserved (CP) samples based on macroscopic samples been explored. This study addresses these inquiries by utilizing a novel, advanced, and fully automated Raman spectrometer, enabling direct perioperative macroscopic sample scanning without any tissue processing such as sectioning or surface manipulation, which would alter the conditions further from the surgical setting. This approach enables the scanning of freshly extracted tumor samples in their native state, facilitating comparisons with scans of cryopreserved and formalin-fixed samples.

In the examination of cryopreserved samples, a general decrease in peak intensity is noted. This implies that cryopreservation does not induce specific biochemical changes in tissue samples; rather, an overall intensity decrease is observed, in line with reduced vibration of molecular bonds with decreased Brownian motion at lower temperatures. Utilizing our SVM classifier, initially trained only on native data, we achieved a classification sensitivity of 75% and a specificity of 100% when applied to cryofixed data. Overall, the initial phase of our study demonstrates that RS, in conjunction with a SVM classifier, can effectively differentiate between the same tissue type when measured in its native and cryopreserved states. These findings hold potential significance, particularly in the context of training a multiclass classifier.

Wills et al. have reported similar findings in their exploration of the potential inclusion of thawed neuropediatric tumors using Raman microscopy to expand the pool of raw spectra [25]. Thereby, the capacity of RS to differentiate between native and frozen samples, along with the preserved ability to identify the specific tumor entity in a frozen sample, has been demonstrated. This supports the notion of incorporating tissue from cryobanks. In our study, we examine various types of neuronal tissues in a cryopreserved state at a macroscopic level. This analysis enables a detailed examination of the impact of cryopreservation on a sample, a crucial aspect for further investigations involving samples extracted from tumor banks.

The lower sensitivity and specificity observed in frozen sections can be attributed to the formation of ice crystals within the sample, leading to the rupture of biochemical structures, cell membrane bursts, and the creation of holes. If a measuring point coincides with such a hole, the spectra exhibit reduced sensitivity due to the absence of tissue at that specific location. This phenomenon, observed and described in frozen histological sections by Raman microscopy by Kalkanis et al. [26], underscores the challenges associated with frozen sections. Despite these issues, they do not overwhelmingly dominate the results. It should be noted that in some instances, we found that the spectral intensity was so low, likely due to this phenomenon in the respective specimen, that it was not possible to extract convincing data, such as in the case of modCNS in Figure 1. This can be a limitation of intraoperative application, where it would be necessary to rely on multiple location sampling as well as additional information sources next to RS.

Our study demonstrates the feasibility of incorporating tumor bank tissues as an existing source of potentially scannable samples without the need for defrosting and consequently destroying the samples. Raman spectroscopy proves its suitability for detecting the tumor’s origin even in the presence of freezing artifacts. Dörr et al. utilized RS to verify damage-free storage of biobank samples, focusing on the OH-stretching band (crystalline/amorphous water content) [20]. A rapid increase in sample numbers will enhance the sensitivity and specificity of the RS machine over a short period, thereby solidifying its position as a powerful tool in tumor diagnostics. Importantly, there is no necessity for specific classifier training on cryofixed data. The demonstrated reversibility of the intensity decrease induced by cryofixation highlights the potential use of tumor bank samples, even if defrosting is required to augment the sample pool.

In the dataset of formalin-fixed samples, distinct peaks were observed, varying among different sample entities and relying on diverse biochemical structures. The SVM classifier, originally trained on native data, demonstrated a differentiation between formalin-fixed meningioma and dura mater samples with a sensitivity of 41.9% and a specificity of 100%. Introducing a different spectrum (obtained by subtracting the spectrum of pure formalin) enhanced these results, yielding a sensitivity of 62.8% and a specificity of 100%. If the alteration in the Raman spectrum of formalin-fixed samples were merely an additive effect without additional chemical reactions, the mathematical procedure of subtraction of the pure formalin spectrum would have been expected to completely mitigate the formalin’s impact. However, this oversimplification overlooks the fact that the Raman intensity at a specific wavenumber is not directly proportional to the number of detected molecular bonds but rather a combination of various molecular vibrations [27]. The complexity of this relationship between native and formalin-fixed samples highlights that the impact of formalin involves a distinct reaction scheme with multiple functional groups, as elucidated in the subsequent discussion.

Several authors have already described the effects of formaldehyde fixation on Raman spectra. As a striking feature, a couple of publishers noted an overall diminution of Raman intensities and even a dramatic decrease in certain characteristic peaks such as 717 cm^−1^, 1637 cm^−1^, 2930 cm^−1^ and 2960 cm^−1^ [18,21,22,23,24]. By applying complementary micro-spectroscopy, Hackett et al. demonstrated that a considerable number of water-soluble species of amino acids, carbohydrates, lipids, phosphates, etc. are leached during the formalin fixation process [24]. O’Faolaoin et al. suspected that functional groups are altered in charge and morphology, especially regarding the amine I band at 1637 cm^−1^ which diminished immensely after fixation [21]. They mentioned the cross-linking reaction between the nitrogen atom of lysine (amide I) and the nitrogen atom of a peptide linkage, generating a tertiary amide. In addition, they postulated protein secondary structure alterations. In contrast, the phenylalanine band at 1003 cm^−1^ is resistant to formaldehyde fixation changes, so it can be used as a reference or normalization band [22]. Furthermore, a series of formalin contamination peaks could be identified: 907 cm^−1^, 1040 cm^−1^, 1254 cm^−1^ and 1490 cm^−1^, resulting from protein conformational changes [21,28]. Another theory is that they may appear due to the unraveling of new functional groups, such as the NH3+ group, after formalin cross-linking reactions [21]. The decrease in the peak ratios of 1445 cm^−1^ /1655 cm^−1^ and 1302 cm^−1^ /1265 cm^−1^ was interpreted as a shift in protein and lipid relations [18]. When examining the spectra of pure formalin, Hackett et al. strangely did not observe a n (C==O) band, representing the essential molecular vibration of formaldehyde. They concluded that the majority of formaldehyde is instantly converted to its equilibrium reaction product, methylene glycol [24]. According to Fiedler et al., formaldehyde fixation-induced spectral changes are principally related to parameters associated with collagen composition [29]. Finally, it is emphasized in the literature that formalin fixation is not an appropriate method to focus on metabolic pathways or disease pathogenesis because the fixing method contributes itself to biochemical changes [24]. Nevertheless, it might find application in diagnostic contexts, a hypothesis explored in the present study.

Furthermore, training a new classifier on the ‘difference spectrum data’ further improved the outcomes, achieving a sensitivity of 66.7% and a specificity of 100%. However, it’s important to note that this approach is more academically oriented, given that these artificial spectra necessitate the final treatment of samples through formalin fixation. Consequently, they are not suitable for in vivo applications but rather serve the purpose of evaluating extensive data from a tumor database.

These findings are particularly noteworthy for dual-class classification, such as distinguishing between healthy and malignant tissues. However, caution is warranted when considering the training of a multiclass classifier on formalin fixed data, as the spectral interference of formalin varies with different biochemical tissues, posing potential challenges. 

Due to our constraints in working with a limited number of tissue samples for both cryofixation and formalin fixation, it is imperative to expand and validate these findings in a larger sample cohort. The inclusion of additional tumor banks and conducting further studies has the potential to reduce this discrepancy, providing a more robust and reliable outcome. 

## 4. Materials and Methods

### 4.1. Raman Spectroscopy

RS is based on different vibrational modes of chemical bonds, which alter according to the biochemical composition of the samples. When the laser source emits a light beam onto a molecule, a small part of the incident photons (on average, one out of 10^7^ photons) loses or gains a certain amount of energy (stokes and anti-stokes effects). These photons will be scattered consecutively due to interaction with different molecular bonds, leading to a specific spectrum of the molecules. Since a sample is generally composed of a huge diversity of biochemical components, the analysis by RS provides a specific spectral footprint of the scanned sample [30,31]. 

### 4.2. Sample Acquisition

For this investigation, we employed a robotized RS-machine (Solais™, Synaptive^®^, Toronto, ON, Canada) featuring a movable stage, a visible light imaging camera (VLI-camera), and a 785 nm laser source at 50 mW power. The samples were measured on an aluminum background chosen for its negligible Raman spectrum, ensuring no interference of the background with the sample spectrum. A coordination system linked to the VLI images enabled the retrospective tracing of the exact measuring point positions. Each spectrum is constructed from 1602 individuals distributed across a wavenumber scale from 314 cm^−1^ to 2994 cm^−1^, amalgamated into a spectral curve through a computerized system. Raman scans were acquired with an illumination time ranging from 800 ms to 2000 ms and 6 to 30 acquisitions for each measuring point.

The project involved the examination of human tissue samples derived from a total of 56 patients (Table 1 and Table 2). The samples were obtained for clinical purposes during neurosurgery at the Centre Hospitalier du Luxembourg (CHL) and ultimately utilized for pathological interpretation. NFPA were obtained during curative transsphenoidal surgery (Munich and Hamburg, Germany), snap-frozen with dry ice, and stored at −80 °C in the Department of Medicine IV, LMU University Hospital. The study was approved by the ethics committee of the LMU Munich (Nr. 152-10), and all patients gave informed consent. 

### 4.3. Sample Fixation

Since we were interested in the influence of different fixation techniques on our tissues, the samples were scanned under different conditions. Native tissue samples were collected directly during neurosurgery, impregnated with physiological water to avoid dehydration, and placed on an aluminum cup positioned on the movable stage of the RS machine. Physiological water was already used during surgery to hydrate the surgical field; therefore, the samples could be considered native. Once the scan was accomplished, sample fixation was performed. We chose the freezing procedure on dry ice for practical reasons; liquid nitrogen would evaporate directly inside the Raman spectroscope because it is impossible to scan through a thermostable container, resulting in partial thawing of the sample during measurement. The aluminum cup holding the tissue sample was placed on a thin layer of dry ice. This handling kept the tissue sample constantly frozen during the whole scanning process, while also avoiding direct contact between the dry ice and the tissue sample. 

The pituitary adenoma samples were first scanned in the frozen state before being thawed and scanned again at room temperature. 

**Figure 6:**
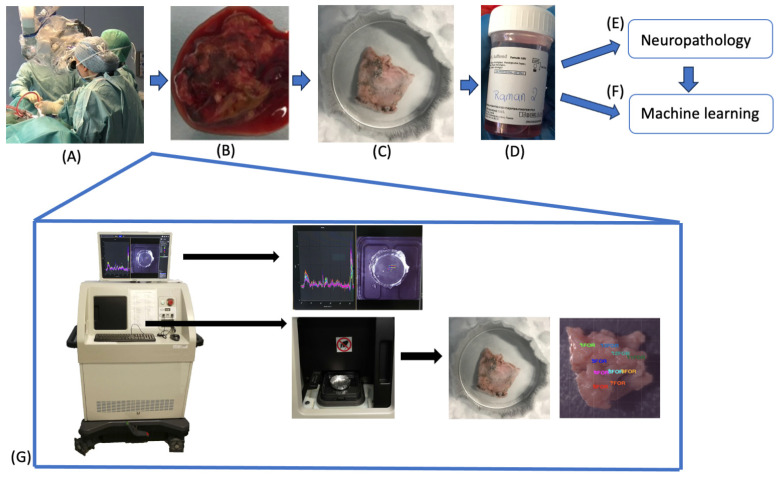
Sample workflow and spectrometer setup. (**A**) Intraoperative sampling. (**B**) First scan in native conditions. (**C**) Second scan in fixed conditions, either cryofixed on dry ice or formalin fixed. (**D**) After the measurement, the samples were stored in formalin solution and (**E**) sent to neuropathology for analysis. (**F**) Every spectrum was attributed with a neuropathological tag according to the results and then used for machine learning. (**G**) Setup of the robotized Raman-spectrometer system (Solais™, Synaptive^®^, Toronto, ON, Canada) with the display of exemplary spectra and the visible light image, where the measuring point locations were defined before (**top row**) as well as the movable stage for the sample placement. For reduction in- background noise, the samples were measured on aluminum dishes, which were put on dry ice for cryofixation purposes (**bottom row**).

In this investigation, the formalin-fixed (FF) samples were stored for varying time durations, ranging from 5 min to several months, in a 4% formaldehyde solution before undergoing scanning. Following the completion of the scanning procedures, all distinct samples were preserved in a formalin solution and dispatched to the LNS (Laboratoire national de santé, Luxembourg) for analysis and interpretation by a neuropathology specialist (Figure 6).

### 4.4. Machine Learning

The gathered data undergoes analysis and correction through dedicated Matlab™ codes and a proprietary graphical user interface called RamanLabeler. The correction process involves correlating retrospective tissue entities with neuropathological diagnoses, sorting spectral outliers, and retracing the precise measuring point locations on the VLI to identify any misscanned points. The average spectra of our scanned samples are scrutinized using a t-SNE (t-distributed stochastic neighbor embedding) algorithm and an SVM classifier (support vector machine classifier) [32,33].

### 4.5. Application of an Internally Trained Classifier

To assess the impact of fixation on the biochemical composition and, consequently, the Raman spectrum of the sample, we employed an internally trained classifier. This classifier is specifically designed to distinguish native dura mater from various meningioma subtypes. Given that the classifier validation has already demonstrated high sensitivity (96.06 ± 0.03%) and specificity (95.44 ± 0.02%) through an internal 5-fold cross-validation and external test set validation (100% sensitivity and 93.97% specificity), we utilized it as conclusive evidence to investigate the extent to which RS can identify meningioma entities in cryopreserved or formalin-fixed samples [8]. Additionally, to enhance classifier performance for formalin fixed sections, a differentiation spectrum was incorporated into the classifier training.

## 5. Conclusions

This article outlines the successful differentiation between fixed and native samples and the accurate retracement of tissue identity following fixation. Specific alternating peaks and a global decrease in peak intensity in cryofixed samples were identified. As CF did not induce specific spectral changes, a classifier trained on native samples can easily be applied to cryofixed samples. However, for formalin fixation, additional experiments are required. Although the use of a difference spectrum (subtracting pure formalin from the spectrum of formalin-fixed samples) improves overall classifier results, it does not yet reach the level of a natively trained classifier used on native or cryofixed data. Training a new classifier based on this difference spectrum also yields improved results.

The ability to analyze an increased number of tissue samples through RS is essential for enhancing machine learning, ultimately leading to the development of an efficient tool for intraoperative and pathological tissue analysis. RS proves to be a promising tool for complementing pathological work quickly and securely without causing tissue destruction. A major limitation in existing studies is the lack of inclusion of rare tumor types, which may only come up once per decade in any given neurosurgery department. Being able to utilize specimens from tumor banks allows for the rapid inclusion of rare tumors as well as large sample numbers for better classification. This paves the way for further studies that delve into the analysis of existing tissue probes, making RS an even more effective and precise diagnostic tool. RS is applicable in both intraoperative settings and retrospective tumor entity analyses, providing a more accurate tumor therapy approach for patients. These factors collectively hold the potential to significantly improve patient outcomes.

## Figures and Tables

**Table 1:** For the sample subset acquired intraoperatively, 407 Raman spectra were acquired from samples of meningioma, dura mater, glioblastoma, or modified CNS tissue, originating from 19 patients. All samples underwent initial measurements in their native state and subsequent measurements in fixed conditions. When calculating the overall patient count, it is crucial to consider the duplicated occurrence of either dura mater, meningioma, or modified CNS tissue samples from the same patients.

Diagnosis	Patients	Sex Ratio (W:M)	Fixation State (Nr of Spectra)			
			Native before FF	Formalin-Fixed (FF)	Native before CF	Cryofixed (CF)
Meningothelial meningioma	4	3:1	13	17	31	21
Dura mater	9	4:5	25	43	24	12
Glioblastoma	8	2:6	49	24	38	22
Modified CNS	7	2:5	32	51	3	2
TOTAL	19	8:11	119	135	96	57

**Table 2:** Overview of the hormone-inactive pituitary adenoma samples taken from the tumor bank. These samples were first scanned in a cryofixed state, then in a thawed condition, and finally heated up to body temperature. In total, 629 spectra were measured.

Diagnosis	Patients	Sex Ratio (W:M)	Fixation State (Nr of Spectra)		
			Cryofixed	Thawed	Body Temperature
Hormone-inactive pituitary adenomas	37	10:27	309	240	80

## Data Availability

The ethical approval does not permit public sharing of data. Interested parties are invited to contact the corresponding author for individual options.

## Supplementary Materials

The following supporting information can be downloaded at: https://www.mdpi.com/article/10.3390/molecules29051167/s1, Figure S1: Raman spectrum of pure substances, first measured natively at room temperature (RT) (red spectrum), then frozen by dry ice (blue spectrum), highlighting the freezing-induced intensity reduction.

## References

[1] André S., Cristau LSaint Gaillard S., Devos O., Calvosa É., Duponchel L. (2015). In-line and real-time prediction of recombinant antibody titer by in situ Raman spectroscopy. Anal. Chim. Acta..

[2] Cho Y., Song S.W., Sung J., Jeong Y.S., Park C.R., Kim H.M. (2017). Hyperspectral depth-profiling with deep Raman spectroscopy for detecting chemicals in building materials. Analyst.

[3] Huang Z., Teh S.K., Zheng W., Mo J., Lin K., Shao X., Ho K.Y., Teh M., Yeoh K.G. (2009). Integrated Raman spectroscopy and trimodal wide-field imaging techniques for real-time in vivo tissue Raman measurements at endoscopy. Opt. Lett..

[4] De Biase D., Paciello O. (2015). Essential and current methods for a practical approach to comparative neuropathology. Folia Morphol..

[5] Bolon B., Anthony D.C., Butt M., Dorman D., Green M.V., Little P.B., Valentine W.M., Weinstock D., Yan J., Sills R.C. (2008). “Current pathology techniques” symposium review: Advances and issues in neuropathology. Toxicol. Pathol..

[6] Fix A.S., Garman R.H. (2000). Practical Aspects of Neuropathology: A Technical Guide for Working with the Nervous System. Toxicol. Pathol..

[7] Boonstra H., Oosterhuis J.W., Oosterhuis A.M., Fleuren G.J. (1983). Cervical tissue shrinkage by formaldehyde fixation, paraffin wax embedding, section cutting and mounting. Virchows Archiv A.

[8] Jelke F., Mirizzi G., Borgmann F.K., Husch A., Slimani R., Klamminger G.G., Klein K., Mombaerts L., Gérardy J.-J., Mittelbronn M. (2021). Intraoperative discrimination of native meningioma and dura mater by Raman spectroscopy. Sci. Rep..

[9] Klamminger G.G., Klein K., Mombaerts L., Jelke F., Mirizzi G., Slimani R., Husch A., Mittelbronn M., Hertel F., Borgmann F.B.K. (2021). Differentiation of Primary CNS Lymphoma and Glioblastoma Using Raman Spectroscopy and Machine Learning Algorithms. Free Neuropathol..

[10] Devpura S., Thakur J.S., Poulik J.M., Rabah R., Naik V.M., Naik R. (2013). Raman spectroscopic investigation of frozen and deparaffinized tissue sections of pediatric tumors: Neuroblastoma and ganglioneuroma. J. Raman Spectrosc..

[11] Fox C.H., Johnson F.B., Whiting J., Roller P.P. (1985). Formaldehyde fixation. J. Histochem. Cytochem..

[12] Blum F. (1893). Der Formaldehyd als Antisepticum. Med. Wochenschau.

[13] Agarwal A. (2000). Semen banking in patients with cancer: 20-year experience. Int. J. Androl..

[14] Argyle C.E., Harper J.C., Davies M.C. (2016). Oocyte cryopreservation: Where are we now?. Hum. Reprod. Update.

[15] Yu Y.-Y. (2010). Significance of biological resource collection and tumor tissue bank creation. World J. Gastrointest. Oncol..

[16] Bauchet L., Rigau V., Mathieu-Daudé H., Figarella-Branger D., Hugues D., Palusseau L., Bauchet F., Fabbro M., Campello C., Capelle L. (2007). French brain tumor data bank: Methodology and first results on 10,000 cases. J. Neurooncol..

[17] Ghita A., Matousek P., Stone N. (2018). Sensitivity of Transmission Raman Spectroscopy Signals to Temperature of Biological Tissues. Sci. Rep..

[18] Huang Z., McWilliams A., Lam S., English J., McLean D., Lui H., Zeng H. (2003). Effect of formalin fixation on the near-infrared Raman spectroscopy of normal and cancerous human bronchial tissues. Int. J. Oncol..

[19] Ockman N. (1958). The infra-red and Raman spectra of ice. Adv. Phys..

[20] Dörr D., Stracke F., Zimmermann H. (2012). Noninvasive quality control of cryopreserved samples. Biopreserv. Biobank..

[21] Ó Faoláin E., Hunter M.B., Byrne J.M., Kelehan P., McNamara M., Byrne H.J., Lyng F.M. (2005). A study examining the effects of tissue processing on human tissue sections using vibrational spectroscopy. Vib. Spectrosc..

[22] Galli R., Uckermann O., Koch E., Schackert G., Kirsch M., Steiner G. (2014). Effects of tissue fixation on coherent anti-Stokes Raman scattering images of brain. J. Biomed. Opt..

[23] Meade A.D., Lyng F.M., Knief P., Byrne H.J. (2007). Growth substrate induced functional changes elucidated by FTIR and Raman spectroscopy in in–vitro cultured human keratinocytes. Anal. Bioanal. Chem..

[24] Hackett M.J., McQuillan J.A., El-Assaad F., Aitken J.B., Levina A., Cohen D.D., Siegele R., Carter E.A., Grau G.E., Hunt N.H. (2011). Chemical alterations to murine brain tissue induced by formalin fixation. Analyst.

[25] Wills H., Kast R., Stewart C., Rabah R., Pandya A., Poulik J., Auner G., Klein M.D. (2009). Raman spectroscopy detects and distinguishes neuroblastoma and related tissues in fresh and (banked) frozen specimens. J. Pediatr. Surg..

[26] Kalkanis S.N., Kast R.E., Rosenblum M.L., Mikkelsen T., Yurgelevic S.M., Nelson K.M., Raghunathan A., Poisson L.M., Auner G.W. (2014). Raman spectroscopy to distinguish grey matter, necrosis, and glioblastoma multiforme in frozen tissue sections. J. Neurooncol..

[27] Carey P.R., Paulo S. (1982). Biochemical Applications of Raman and Resonance Raman Spectroscopies.

[28] Movasaghi Z., Rehman S., Rehman I.U. (2007). Raman Spectroscopy of Biological Tissues. Appl. Spectrosc. Rev..

[29] Fiedler I.A.K., Casanova M., Keplinger T., Busse B., Müller R. (2018). Effect of short-term formaldehyde fixation on Raman spectral parameters of bone quality. J. Biomed. Opt..

[30] Ferraro J.R., Nakamoto K., Brown C.W. (2003). Introductory Raman Spectroscopy.

[31] Zhang Y., Hong H., Weibo C. (2010). Imaging with Raman spectroscopy. Curr. Pharm. Biotechnol..

[32] Van der Maaten L., Hinton G. (2008). Visualizing Data using t-SNE. J. Mach. Learn. Res..

[33] Benhur A., Weston J., Carugo O., Eisenhaber F. (2009). A User’ s Guide to Support Vector Machines.

[34] Association W.M. (1964). WMA Declaration of Helsinki—Ethical Principles for Medical Research involving Human Subjects [Internet]. https://www.wma.net/policies-post/wma-declaration-of-helsinki-ethical-principles-for-medical-research-involving-human-subjects/.

[35] The Council of the European Union (2014). (Text with EEA Relevance), 2016.

