# Supplementary material for: Impact of Formalin- and Cryofixation on Raman Spectra of Human Tissues and Strategies for Tumor Bank Inclusion
Source: Molecules. 2024 Mar 6;29(5):1167. doi: 10.3390/molecules29051167 (PMC10935185; doi:10.3390/molecules29051167)
Supplement: Supplementary file 1 [file molecules-29-01167-s001.zip › molecules-2870088-supplementary.pdf]

Supplementary Materials

Impact of Formalin- and Cryofixation on Raman Spectra of Human Tissues and Strategies for Tumor Bank Inclusion

Giulia Mirizzi [1,2,†], Finn Jelke [1,2,3,†], Michel Pilot [4], Karoline Klein [2], Gilbert Georg Klamminger [5,6], Jean-Jacques Gérardy [6,7], Marily Theodoropoulou [4], Laurent Mombaerts [1,8], Andreas Husch [8], Michel Mittelbronn [3,6,7,8,9], Frank Hertel [1,2] and Felix Bruno Kleine Borgmann [2,3,10,*]

1 National Department of Neurosurgery, Centre Hospitalier de Luxembourg (CHL), 1210 Luxembourg, Luxembourg
2 Saarland University Medical Center and Faculty of Medicine, 66421 Homburg, Germany
3 Department of Cancer Research (DoCR), Luxembourg Institute of Health (LIH), 1445 Strassen, Luxembourg
4 Department of Medicine IV, LMU University Hospital, LMU Munich, 80539 Munich, Germany
5 Department of General and Special Pathology, Saarland University Medical Center (UKS), Saarland University (USAAR), 66424 Homburg, Germany
6 National Center of Pathology (NCP), Laboratoire National de Santé (LNS), 3555 Dudelange, Luxembourg
7 Luxembourg Center of Neuropathology (LCNP), 3555 Dudelange, Luxembourg
8 Luxembourg Centre for Systems Biomedicine (LCSB), University of Luxembourg (UL), 4365 Esch-sur-Alzette, Luxembourg
9 Department of Life Science and Medicine (DLSM), University of Luxembourg (UL), 4365 Esch-sur-Alzette, Luxembourg
10 Hôpitaux Robert Schuman, 2540 Luxembourg, Luxembourg
* Correspondence: felix.kleineborgmann@lih.lu
† These authors contributed equally to this work.

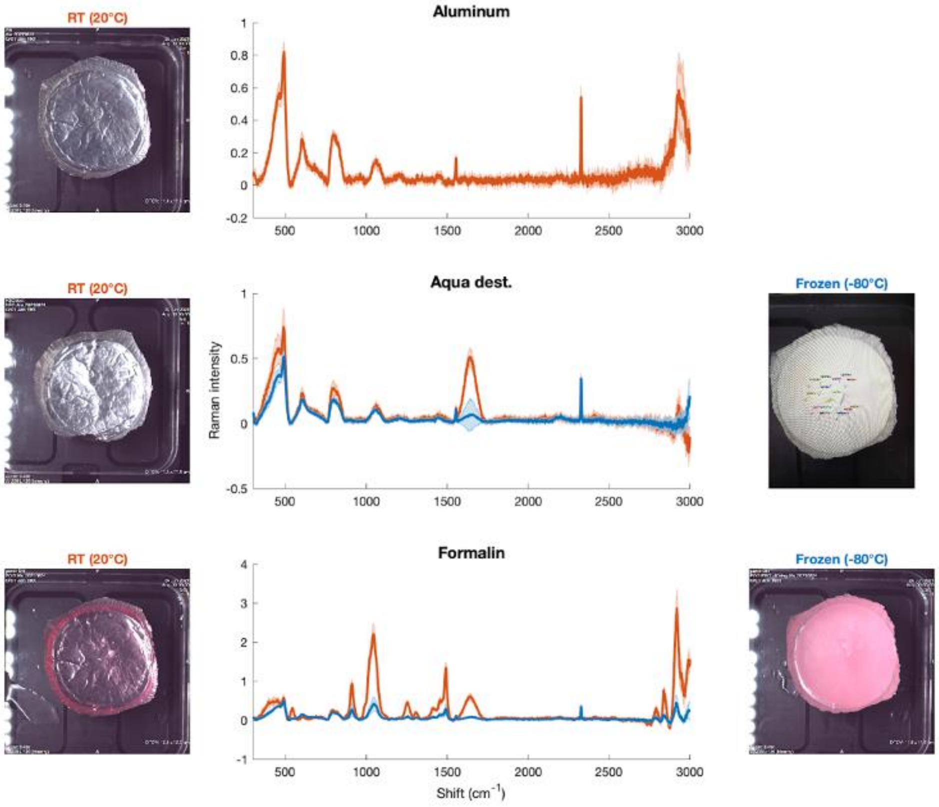

Figure S1. Raman spectrum of pure substances, firstly measured natively at room temperature (RT) (red spectrum), then frozen by dry ice (blue spectrum), highlighting the freezing-induced intensity reduction.
